# On the Immediate Cause of Stammering, and the Means of Cure

**Published:** 1846-05

**Authors:** Henry Butterfield

**Affiliations:** Cantab


*On the Immediate cause of Stammering, and the means of Cure.* By Henry Butterfield, M. A., Cantab.—Having been engaged for some years in the treatment of stammering and defective speech, I propose to notice, in The Lancet, some of the practical results of attentive observation and experiment. Upon the remote cause of stammering I produce no opinion ; but I confine myself to the immediate cause. Much has been written upon the question—"why one person should stammer, and another speak fluently?" But from inattention to an important rule of Sir Isaac Newton, "that no more causes of phenomena are to be admitted than are necessary to explain them," little good has resulted to the public from such inquiries. Would it help us to teach French or Italian, by inquiring into the origin of different languages being spoken? Is not the proper mode that which analyzes the difference between our natural law of speech and the natural law of speech in others? This point must surely be conceded. Now I have contented myself with this process in cases of stammering and defective speech. The result of continued observation enables me to state,—1. That stammerers try to form a note of sound with a wrong position of the lips or tongue. 2. That the vocal chords of the throat then refuse to act, as the effort is made contrary to the natural law of speech. 3. That bad habits and propensities of the nerves and muscles of speech, producing all the phenomena of stammering, soon follow a few wrong efforts to sound with improper position.

It is important to notice here the difference between a child's speaking with a wrong position of the tongue or lips, and a stammerer's trying to speak so. The child, very quietly and unconsciously, combines the vowel of the syllable, whether the consonant position has been right or not,—thus, it says t for k, in the word key; d for g, in the word game. But the stammerer does not combine the vowal with the consonant; he will produce numerous notes of sound, but no vowel will follow in combination, as in the case of the child's prattle; until, with great effort, he has, by chance, approximated to right position, or until he has attained it: then, out bursts the vowel of the syllable, and several syllables or words continuously follow.

The practice or experiment connected with these observations is extremely simple, although it must be specially adapted to every individual case.

I endeavour to produce the first intention of cure by alienating from the bad habits and propensities of the nerves and muscles of speech. This is done by my teaching the method of respiration used by the best professional singers and speakers; and by the use of an alphabet formed to act upon the attention of the mind and the vocal chords of the throat at the same instant.

The subsequent training is derived from the nature of accent and from the grammatical combination of words in a sentence. I teach the stammerer to think and speak his sentences in grammatical parts, and show him that good accentual speaking produces the greatest distinctness and force upon the ear with the greatest ease to the throat, lungs, and organs of respiration. And with this method carried on to the practice of extempore narration, I find that even those who "creep unwillingly to school," and puzzle their tutors, by stammering wilfully with the mouth, to hide the stammering of the mind, can be made with certainty to read and speak properly when they please.

My engagement for some years as extra master, at Eton, for the treatment of stammering and defective speech, has enabled me to pursue my course of observation and experiment with the greatest advantage; and several eminent surgeons have recommended me to extend my treatment of this calamity, in conjunction with their medical or surgical treatment, when required.—*Lond. Lancet.*

